# Full-Length Transcriptome and RNA-Seq Analyses Reveal the Mechanisms Underlying Waterlogging Tolerance in Kiwifruit (*Actinidia valvata*)

**DOI:** 10.3390/ijms23063237

**Published:** 2022-03-17

**Authors:** Zhi Li, Danfeng Bai, Yunpeng Zhong, Miaomiao Lin, Leiming Sun, Xiujuan Qi, Chungen Hu, Jinbao Fang

**Affiliations:** 1 Key Laboratory for Fruit Tree Growth, Development and Quality Control, Zhengzhou Fruit Research Institute, Chinese Academy of Agricultural Sciences, Zhengzhou 450009, China; lizhizzgss21@163.com (Z.L.); baidanfeng1993@163.com (D.B.); linmiaomiao@caas.cn (M.L.); sleiming@163.com (L.S.); qixiujuangaoxing@163.com (X.Q.); 2 Key Laboratory of Horticultural Plant Biology, College of Horticulture & Forestry Sciences of Huazhong Agricultural University, Wuhan 430070, China; chungen@mail.hzau.edu.cn

**Keywords:** *Actinidia valvata*, waterlogging, carbohydrate, free amino acid, oxidative stress, transcription factor

## Abstract

*Actinidia valvata* possesses waterlogging tolerance; however, the mechanisms underlying this trait are poorly characterized. Here, we performed a transcriptome analysis by combining single-molecule real-time (SMRT) sequencing and Illumina RNA sequencing and investigated the physiological responses of the roots of KR5 (*A. valvata*, a tolerant genotype) after 0, 12, 24 and 72 h of waterlogging stress. KR5 roots responded to waterlogging stress mainly via carbohydrate and free amino acids metabolism and reactive oxygen species (ROS) scavenging pathways. Trehalose-6-phosphate synthase (TPS) activity, alcohol dehydrogenase (ADH) activity and the total free amino acid content increased significantly under waterlogging stress. The nicotinamide adenine dinucleotide-dependent glutamate synthase/alanine aminotransferase (NADH-GOGAT/AlaAT) cycle was correlated with alanine accumulation. Levels of genes encoding peroxidase (POD) and catalase (CAT) decreased and enzyme activity increased under waterlogging stress. Members of the LATERAL ORGAN BOUNDARIES (LOB), AP2/ERF-ERF, Trihelix and C3H transcription factor families were identified as potential regulators of the transcriptional response. Several hub genes were identified as key factors in the response to waterlogging stress by a weighted gene co-expression network analysis (WGCNA). Our results provide insights into the factors contributing to waterlogging tolerance in kiwifruit, providing a basis for further studies of interspecific differences in an important plant trait and for molecular breeding.

## 1. Introduction

Waterlogging stress negatively affects plant growth and development [1]. In waterlogged soil, root tissues are submerged in water, causing a rapid decline in the oxygen concentration around the root rhizosphere. Hypoxia limits aerobic respiration in roots, resulting in a lack of energy [2]. Plants also face other challenges, such as cytoplasmic acidosis, nutrient deficiency, oxidative stress, and toxic chemicals accumulation, and develop chlorosis, wilting, die-back and rotting symptoms under waterlogging stress [3,4,5,6]. To survive waterlogging stress, they have evolved various strategies including the formation of aerenchyma and adventitious roots at the morphological level [7] and molecular responses, as determined by RNA-Seq in *Arabidopsis* [8], cotton [9], sesame [10], and other plants. Increasing research is focused on the roles of key pathways involved in adaption to waterlogging stress [11,12].

Plants produce adenosine triphosphate (ATP) mainly from anaerobic fermentation under waterlogging stress [13]. Genes encoding enzymes involved in glycolysis and fermentation are upregulated [14,15,16], while genes involved in energy-consuming process, such as protein and DNA synthesis, are usually downregulated under hypoxic conditions [17,18]. The dynamics of carbohydrates, such as sucrose and glucose, are altered under waterlogging in plants [19,20]. Free amino acid metabolism is associated with plant stress adaptation [21,22]. Amino acids act as reactive oxygen species (ROS) scavengers or alternative substrates under stress conditions [23]. Waterlogging stress causes the overproduction of ROS, such as superoxide anions and hydrogen peroxide (H_2_O_2_), which leads to lipid peroxidation [24]. Plants have evolved an enzymatic defense system, including superoxide dismutase (SOD), peroxidase (POD) and catalase (CAT) to mitigate these adverse effects [25,26]. The capacity to scavenge ROS is correlated with waterlogging tolerance in plants. The roles of transcription factors (TFs), including the ethylene response factor (ERF), MYB, and basic helix–loop–helix (bHLH) TF families, in waterlogging tolerance in plants have also been described [27,28,29]. 

*A. chinensis* var. *deliciosa* genotypes are widely used as rootstocks for kiwifruit production. De novo transcriptome sequencing of ‘Jinkui’ (*A. deliciosa*, hexaploid) has previously revealed that waterlogging-induced DEGs are enriched in ‘ribosome,’ ‘plant hormone signal transduction,’ and ‘starch and sucrose metabolism’ pathways [30]. Recent studies have shown that tolerance to waterlogging stress is much higher in *A. valvata* than in *A. chinensis* var. *deliciosa* [31]. Accordingly, *A. valvata* genotypes are candidate resources for rootstock selection in kiwifruit. However, a comprehensive understanding of the mechanisms underlying waterlogging tolerance in *A. valvata* is lacking. Genome data are not available for hexaploid kiwifruit. SMRT sequencing enables the acquisition of full-length mRNA [32] and has been combined with RNA-Seq to evaluate the responses to abiotic stresses in species without genome data [33,34]. In this study, transcript-level responses of KR5 (*A. valvata*, hexaploid) roots under waterlogging stress were explored using second- and third-generation sequencing technologies. We also investigated the root physiological responses related to carbohydrate and free amino acid metabolism and ROS detoxification. Our results provide better understanding of why *A. valvata* plants are well-adapted to waterlogging stress.

## 2. Results

### 2.1. Overview of Sequencing Data

**Figure 1:**
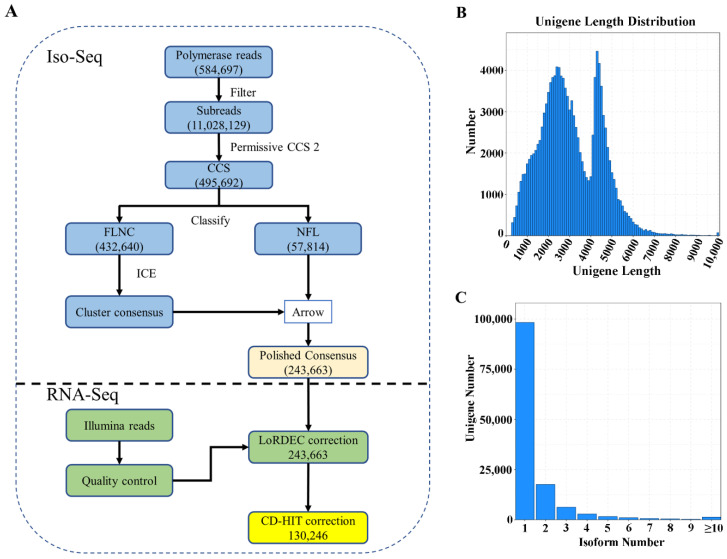
Data processing (**A**) and summary statistics of the unigenes obtained by PacBio Iso-Seq (**B**,**C**). (**A**) overview of the data process; (**B**) length distribution of unigenes; (**C**) isoform number.

Based on SMRT sequencing, 584,697 polymerase reads, 11,028,129 subreads and 495,692 circular consensus sequences (CCSs) were obtained (Figure 1A, Table S1). From the CCSs, 432,640 sequences were identified as full-length non-chimeric (FLNC) reads. In total, 243,663 polished consensus isoforms were generated. After error correction and redundancy removal, 130,246 unigenes were obtained, with an N50 of 3803 bp and a mean length of 3025 bp (Table S1). Among the unigenes, 7083, 24,620 and 37,217 were 0.5–1, 1–2, 2–3 kb, respectively (Figure 1B). In addition, 98,303 unigenes had only one transcript (Figure 1C). Based on benchmarking universal single-copy ortholog (BUSCO) analysis, 394 (91.6%) of the 430 expected embryophyte genes were identified as complete after removing redundancy (Figure S1). The high percentage of complete BUSCO genes indicated high integrity for subsequent analysis. Illumina RNA-Seq generated 20.5–27.1 million raw reads and 20.0–26.7 million clean reads for each root sample (Table S2). The Q30 percentages were 82.50–95.78% and the GC contents were 45.03–47.60%. 

### 2.2. Gene Annotation and Functional Classification

Among 130,246 unigenes, 123,863 (95.10%) unigenes were annotated using the NR database (Figure S2A) and 54,850 (42.12%) unigenes were annotated using five public databases (Figure S2B). In a Gene Ontology (GO) enrichment analysis, the top three enriched GO terms in the biological process category were ‘metabolic process’, ’cellular process’, and ‘single-organism process’. In the cellular component category, the top three enriched GO terms were ‘cell’, ‘cell part’, and ‘organelle’. In the molecular function category, the top three enriched GO terms were ‘binding’, ‘catalytic activity’, and ‘transporter activity’ (Figure S2C). 

### 2.3. Statistical Summary of DEGs

**Figure 2:**
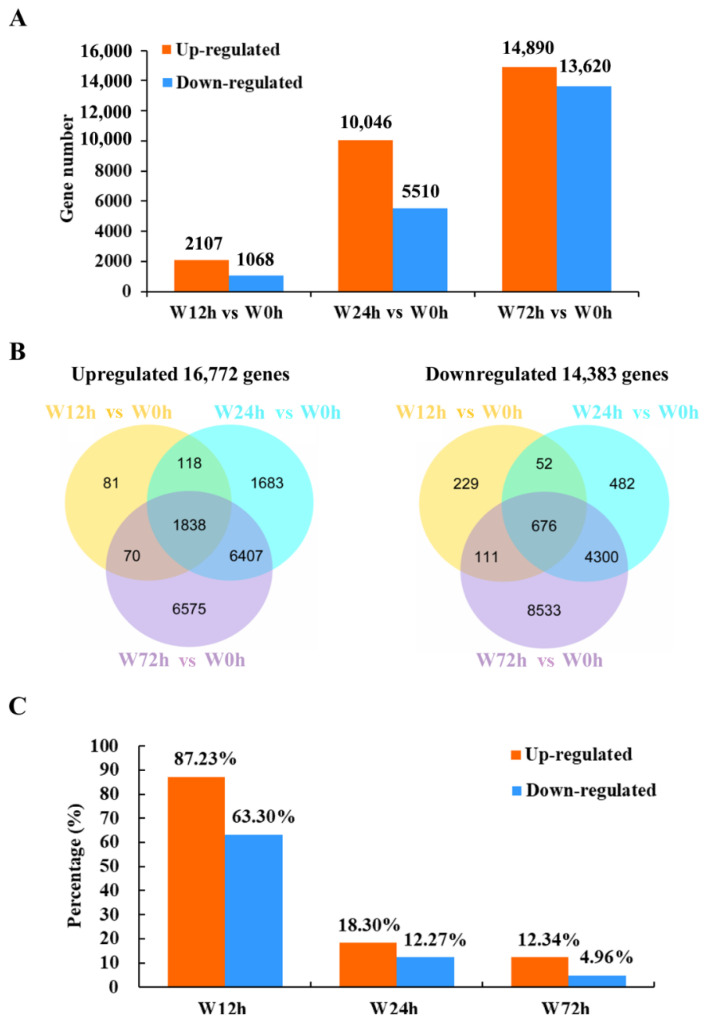
Summary of DEGs after different periods of waterlogging. (**A**) number of DEGs; (**B**) venn diagram of the number of DEGs after different durations of waterlogging; (**C**) percentage of the number of co-expressed DEGs to the number of DEGs induced after different durations of waterlogging.

The numbers of up- and down-regulated DEGs between the control (0 h of waterlogging) and waterlogging treatments increased gradually over time (Figure 2A). There were 1838 and 676 commonly up- and down-regulated DEGs, respectively, among different periods of waterlogging stress (Figure 2B). Additionally, 310 (81 up- and 229 down-regulated), 2165 (1683 up- and 482 down-regulated) and 15,108 (6575 up- and 8533 down-regulated) DEGs were specifically expressed after 12, 24 and 72 h of waterlogging, respectively. The percentages of co-expressed upregulated and downregulated DEGs were highest after 12 h of waterlogging (Figure 2C). These results indicated that a conserved transcript adjustment was stimulated at as early as 12 h of waterlogging and dynamic transcriptomic changes were induced after 24 or 72 h of waterlogging stress in KR5 roots.

### 2.4. KEGG Analysis of Co-Expressed DEGs

**Figure 3:**
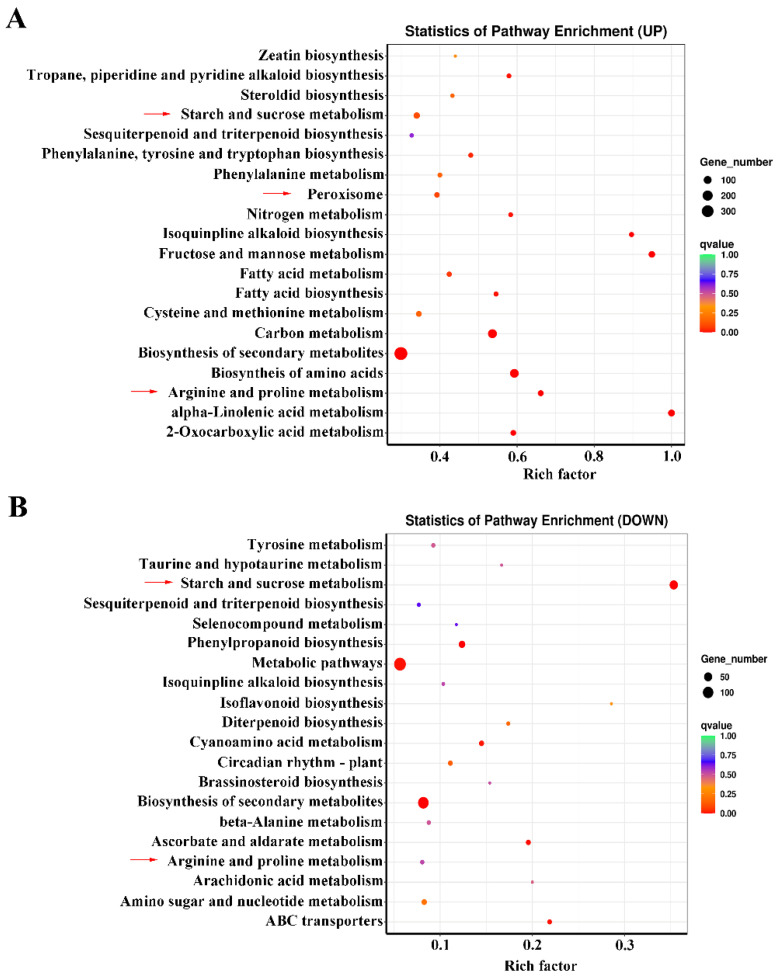
KEGG analysis of co-expressed DEGs. (**A**) enriched pathways for the upregulated DEGs; (**B**) enriched pathways for the downregulated DEGs.

In a KEGG pathway enrichment analysis of co-expressed up-regulated DEGs, the most significant pathways included ‘starch and sucrose metabolism’, ‘fructose and mannose metabolism’, ‘arginine and proline metabolism’, ‘biosynthesis of amino acid’, ‘nitrogen metabolism’, and ‘peroxisome’ (Figure 3A). For the co-expressed down-regulated DEGs, the ‘starch and sucrose metabolism’, ‘arginine and proline metabolism’, and ‘beta-alanine metabolism’ pathways were significantly enriched (Figure 3B). These results showed that the responses of KR5 roots to different durations of waterlogging stress mainly involved the regulation of carbohydrate and amino acid metabolisms and ROS scavenging.

Additionally, 29 co-expressed upregulated DEGs were enriched in ‘fatty acid metabolism’ and ‘fatty acid biosynthesis’ and ‘alpha-linolenic acid metabolism’ pathways (Figure 3A). A heatmap analysis of all the 29 DEGs showed that expression levels of 11 were much more highly promoted under waterlogging stress than others (Figure S3). The 11 significantly upregulated DEGs included 4 DEGs encoding long-chain acyl-CoA synthetase (LACS), 5 DEGs encoding stearoyl-l ACP desaturase (SAD), 1 DEG encoding acetyl-CoA C-acetyltransferase (AACT) and 1 DEG encoding lipoxygenase (LOX) (Figure S3). It seemed that fatty acid and lipid metabolisms also played a role in the response to waterlogging stress in KR5 roots.

**Figure 4:**
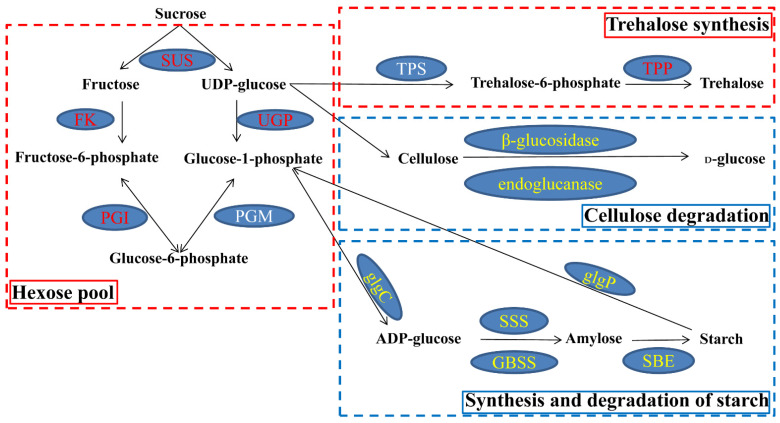
Co-expressed DEGs involved in the ‘starch and sucrose metabolism’ pathway. Transcript levels of DEGs encoding enzymes that increased during waterlogging stress are shown in red; transcript levels of DEGs encoding enzymes that decreased are shown in yellow. One of two DEGs encoding TPS was up-regulated under waterlogging stress. No co-expressed DEGs encoding phosphoglucomutase (PGM) were enriched in the ‘starch and sucrose metabolism’ pathway. Abbreviations are as follows: FK, fructokinase; UGP, UTP-glucose-1-phosphate uridylyl-transferase; PGI, glucose-6-phosphate isomerase; TPP, trehalose-phosphate phosphatase; glgC, ADP-glucose pyro-phosphorylase; glgP: alpha-1,4 glucan phosphorylase; SSS: soluble starch synthase; GBSS, granule-bound starch synthase; SBE, starch-branching enzyme.

In the ‘starch and sucrose metabolism’ pathway, there were 51 and 53 commonly upregulated and downregulated DEGs, respectively. The upregulated DEGs encoding SUS, fructokinase (FK), hexokinase (HK), UTP-glucose-1-phosphate uridylyltransferase (UGP), glucose-6-phosphate isomerase (PGI), TPS and trehalose-phosphate phosphatase (TPP) were involved in hexose and trehalose metabolism (Figure 4 and Figure S4A). The downregulated DEGs encoding β-glucosidase, endoglucanase, alpha-1,4 glucan phosphorylase (glgP), ADP-glucose pyro-phosphorylase (glgC), soluble starch synthase (SSS), granule-bound starch synthase (GBSS) and starch-branching enzyme (SBE) were involved in the degradation of cellulose and starch metabolism (Figure 4 and Figure S4B). These results indicated that carbohydrate metabolism was profoundly adjusted under waterlogging stress in KR5 roots at the transcript level. 

### 2.5. Carbohydrate Metabolism under Waterlogging Stress

**Figure 5:**
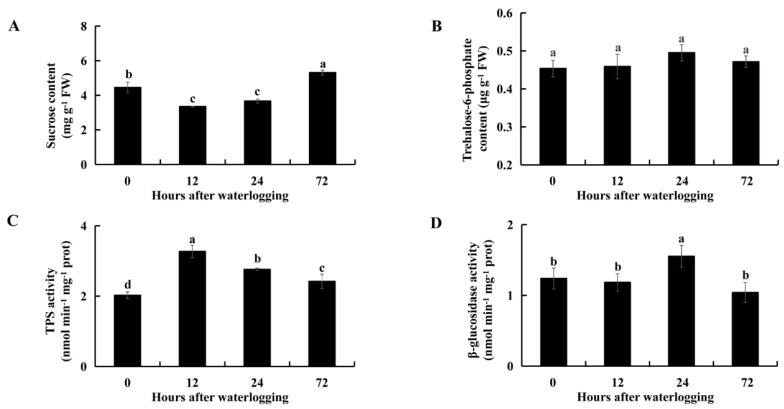
Changes in sucrose (**A**) and T6P (**B**) contents, as well as TPS (**C**) and β-glucosidase (**D**) activity in KR5 roots under waterlogging stress. Data are expressed as averages ± SD, *n* = 3. Significant differences between treatments at *p* < 0.05 are indicated by different letters over columns (Duncan’s test).

Compared with levels in controls, the sucrose contents were significantly lower after 12 h (*p* = 7.1 × 10^−4^) and 24 h (*p* = 6.8 × 10^−3^) of waterlogging stress and were significantly higher after 72 h (*p* = 3.7 × 10^−3^) of waterlogging in KR5 roots (Figure 5A). Sucrose degradation provides substrates for the synthesis of trehalose-6-phosphate (T6P), a process that is catalyzed by TPS. The T6P content was slightly higher in the KR5 roots under waterlogging stress than in the controls (Figure 5B). Interestingly, TPS activity was significantly higher in KR5 roots under waterlogging stress than in controls (*p* < 0.05, Figure 5C). β-glucosidase is an enzyme responsible for cellulose degradation. The activity of β-glucosidase was slightly lower after 12 or 72 h of waterlogging than in controls and significantly higher after 24 h (*p* = 0.027) of waterlogging in KR5 roots. The above results revealed that KR5 roots responded to waterlogging by the regulation of sucrose, T6P and cellulose metabolism.

### 2.6. Ethanolic Fermentation under Waterlogging Stress

**Figure 6:**
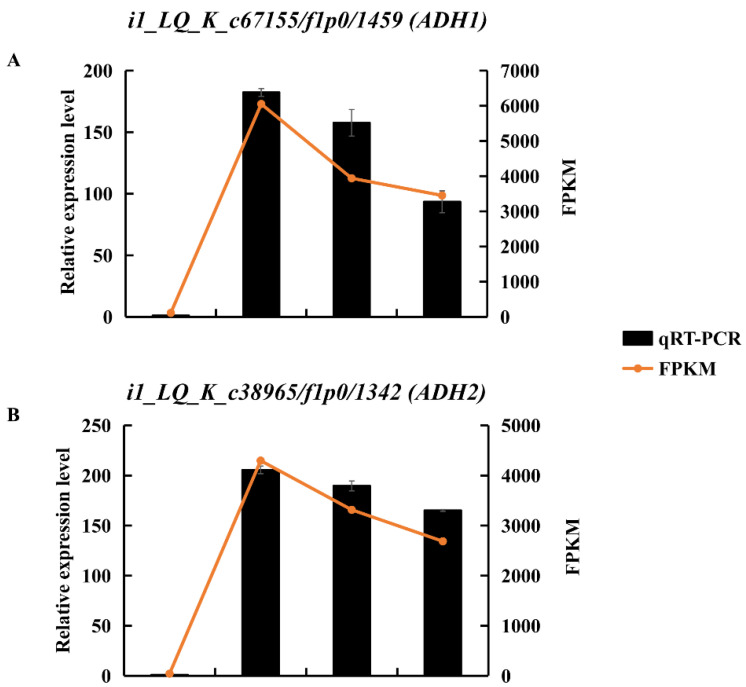

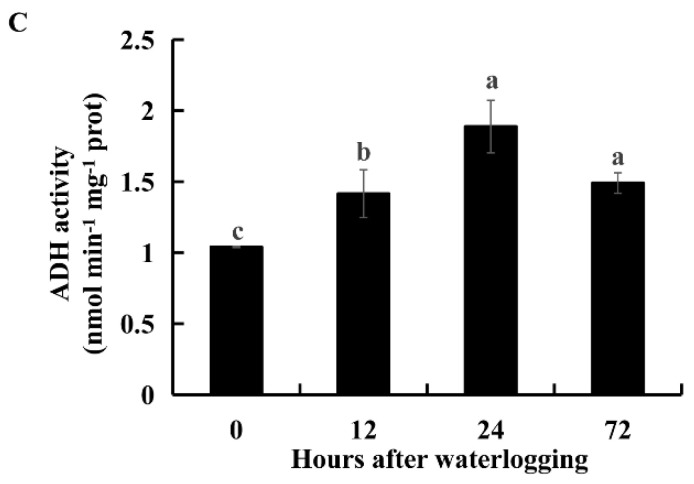
Changes of expression levels of two DEGs encoding ADH and ADH activity in KR5 roots under waterlogging stress. (**A**) RNA-Seq (right vertical axis) and qRT-PCR (left vertical axis) results for transcript changes in *i1_LQ_K_c67155/f1p0/1459* (*ADH1*); (**B**) RNA-Seq (right vertical axis) and qRT-PCR (left vertical axis) results for transcript changes in *i1_LQ_K_c38965/f1p0/1342* (*ADH2*); (**C**) ADH activity. In (**C**) data are expressed as averages ± SD, *n* = 3 and significant differences between treatments at *p* < 0.05 are indicated by different letters over columns (Duncan’s test).

ADH is a marker of anaerobic fermentation. Transcript levels of DEGs encoding ADHs were upregulated in KR5 roots under waterlogging stress (Figure S5). RNA-Seq results showed that the expression levels of *i1_LQ_K_c67155/f1p0/1459* (*ADH1*) and *i1_LQ_K_c38965/f1p0/1342* (*ADH2*) were most significantly upregulated under waterlogging stress (Figure 6A,B and Figure S5). The upregulation of these two *ADHs* under waterlogging stress was validated by qRT-PCR (Figure 6A,B). ADH activity was significantly higher under waterlogging stress than in the controls (*p* < 0.05, Figure 6C). These results indicated that ethanolic fermentation was activated in KR5 roots under waterlogging stress.

### 2.7. Free Amino Acid Metabolism under Waterlogging Stress

**Figure 7:**
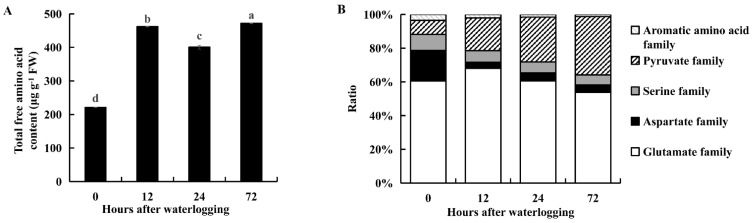
Changes of the total free amino acid content (**A**) and the proportion of different amino acid family (**B**) in KR5 roots under waterlogging stress. In (**A**) the total free amino acid content was estimated as the sum of the contents of 16 individual free amino acids. Data are expressed as averages ± SD, *n* = 3. Significant differences between treatments at *p* < 0.05 are indicated by different letters over columns (Duncan’s test).

The contents of the major amino acids increased under waterlogging stress (Table S3). The levels of glutamate, alanine and arginine were higher than those of the other amino acids (Table S3). The total content of 16 individual amino acids was significantly higher under waterlogging stress than in the controls (*p* < 0.05, Figure 7A). The proportion of amino acids belonging to the glutamate family increased after 12 or 24 h of waterlogging and later decreased after 72 h of waterlogging (Figure 7B). The proportion of amino acids belonging to the pyruvate family increased during 72 h of waterlogging stress (Figure 7B). The proportion of amino acids belonging to the aspartate, serine and aromatic amino acid families all decreased during 72 h of waterlogging stress (Figure 7B). Thus, KR5 roots responded to waterlogging stress by modulating the levels of free amino acids.

### 2.8. Enzymes and DEGs Related to Alanine and Glutamate Metabolism

**Figure 8:**
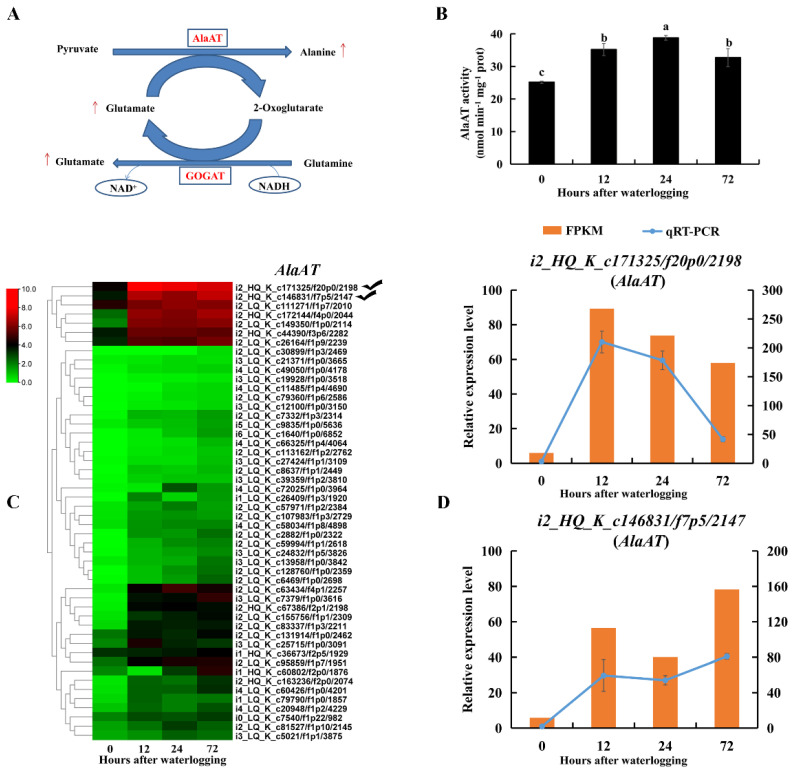
Changes in transcript level and enzyme activity of AlaAT in KR5 roots under waterlogging stress. (**A**) alanine and glutamate synthesis. (**B**) changes of the activity of AlaAT. (**C**) transcript levels of *AlaATs*. (**D**) RNA-Seq (right vertical axis) and qRT-PCR (left vertical axis) results for transcript changes in two *AlaATs* which were highly induced under waterlogging stress. In (**B**) data are expressed as averages ± SD, *n* = 3 and significant differences between treatments at *p* < 0.05 are indicated by different letters over columns (Duncan’s test).

There was a 14-fold increase in the amount of free alanine in roots after 12 h of waterlogging compared to levels in the controls (Table S3). AlaAT catalyzes the formation of alanine by consuming pyruvate and glutamate. AlaAT activity was significantly increased in KR5 roots under waterlogging stress compared to levels in the controls (*p* < 0.05, Figure 8B). Transcript levels of AlaATs encoded DEGs were mostly upregulated under waterlogging stress (Figure 8C). RNA-Seq and qRT-PCR results all showed that transcript levels of *i2_HQ_K_c171325/f20p0/2198* and *i2_HQ_K_c146831/f7p5/2147* were highly promoted under waterlogging stress (Figure 8D).

**Figure 9:**
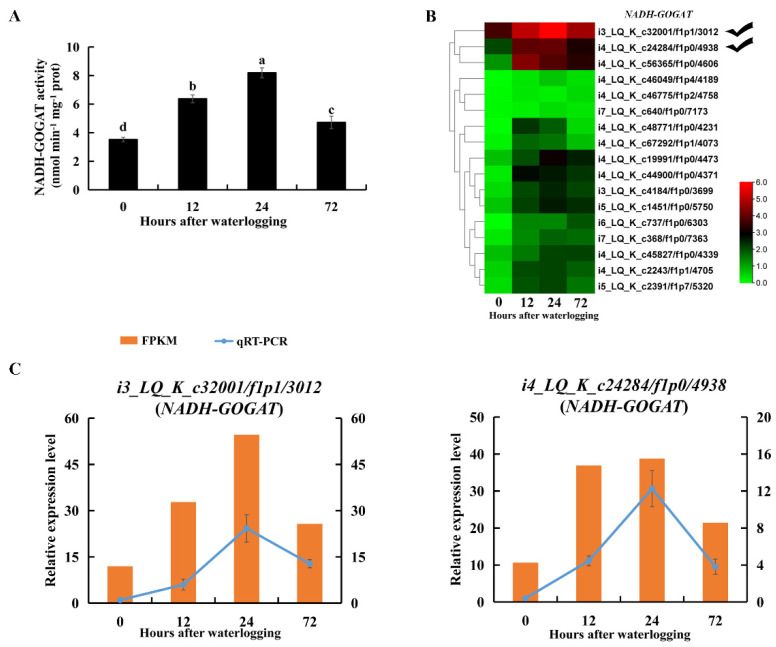
Changes in transcript level and enzyme activity of NADH-GOGAT in KR5 roots under waterlogging stress. (**A**) changes of the activity of NADH-GOGAT; (**B**) transcript changes of *NADH-GOGATs*; (**C**) RNA-Seq (right vertical axis) and qRT-PCR (left vertical axis) results for transcript changes in two *NADH-GOGATs* which were highly induced under waterlogging stress. In (**A**) data are expressed as averages ± SD, *n* = 3 and significant differences between treatments at *p* < 0.05 are indicated by different letters over columns (Duncan’s test).

The glutamate content was also significantly higher under waterlogging stress than in the control (*p* < 0.05, Table S3). NADH-GOGAT catalyzes the formation of glutamate by transferring the glutamine amide group to 2-oxoglutarate. NADH-GOGAT activity was significantly enhanced in roots during 72 h of waterlogging stress (*p* < 0.05, Figure 9A). Transcript levels of DEGs encoding NADH-GOGAT were also upregulated under waterlogging stress (Figure 9B). RNA-Seq and qRT-PCR results all showed that transcript levels of *i3_LQ_K_c32001/f1p1/3012* and *i4_LQ_K_c24284/f1p0/4938* were highly promoted under waterlogging stress (Figure 9C). These results indicated that the NADH-GOGAT/AlaAT cycle was correlated with alanine and glutamate metabolism in KR5 roots under waterlogging stress.

### 2.9. Enzymes and DEGs Related to ROS Scavenging

**Figure 10:**
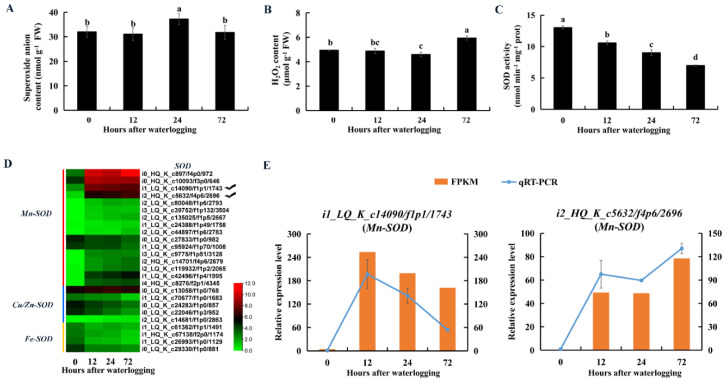
Changes in contents of superoxide anion and hydrogen peroxide and activity of superoxide dismutase (SOD) and expression patterns of *SODs* in KR5 roots under waterlogging stress. (**A**) superoxide anion content; (**B**) H_2_O_2_ content; (**C**) SOD activity; (**D**) transcript levels of *SODs*; (**E**) RNA-Seq (right vertical axis) and qRT-PCR (left vertical axis) results for transcript changes in two *Mn-SODs* which were highly induced under waterlogging stress. In (**A**–**C**) data are expressed as averages ± SD, *n* = 3 and significant differences between treatments at *p* < 0.05 are indicated by different letters over columns (Duncan’s test).

**Figure 11:**
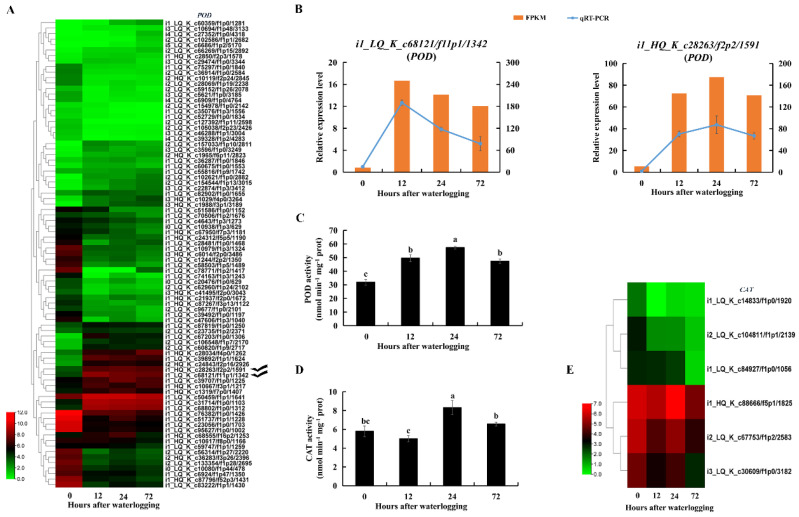
Changes in contents of hydrogen peroxide and superoxide anion and activities of peroxidase (POD) and catalase (CAT) and expression patterns of *PODs* and *CATs* in KR5 roots under waterlogging stress. (**A**) transcript levels of *PODs*; (**B**) RNA-Seq (right vertical axis) and qRT-PCR (left vertical axis) results for transcript changes in two *PODs* which were highly induced under waterlogging stress; (**C**) POD activity; (**D**) CAT activity; (**E**) transcript levels of *CATs*. In (**C**,**D**) data are expressed as averages ± SD, *n* = 3 and significant differences between treatments at *p* < 0.05 are indicated by different letters over columns (Duncan’s test).

The superoxide anion content was significantly higher in KR5 roots after 24 h of waterlogging than in controls (Figure 10A). SOD catalyzes the dismutation of superoxide anions into H_2_O_2_. Total SOD activity was significantly decreased in KR5 roots within 72 h of waterlogging (*p* < 0.05, Figure 10C). At the transcript level, DEGs encoding manganese SOD (Mn-SOD) were mostly up-regulated, while DEGs encoding copper-zinc and iron SODs were down-regulated under waterlogging stress (Figure 10D). Both RNA-Seq and qRT-PCR results showed that transcript levels of two *Mn-SODs* (*i1_LQ_K_c14090/f1p1/1743* and *i2_HQ_K_c5632/f4p6/2696*) were highly promoted under waterlogging stress (Figure 10E). POD and CAT can reduce H_2_O_2_ into water and oxygen. The H_2_O_2_ content decreased slightly after 12 and 24 h of waterlogging and then increased significantly after 72 h of waterlogging compared with levels in controls (*p* = 8.4 × 10^−4^, Figure 10B). POD activity increased significantly within 72 h of waterlogging (*p* < 0.05, Figure 11C). At the transcript level, DEGs encoding POD were mostly down-regulated under waterlogging stress (Figure 11A). However, both RNA-Seq and qRT-PCR results showed that transcript levels of two *PODs* (*i1_LQ_K_c68121/f11p1/1342* and *i1_HQ_K_c28263/f2p2/1591*) were still highly promoted under waterlogging stress (Figure 11B). CAT activity increased significantly after 24 h of waterlogging (*p* = 3.2 × 10^−3^, Figure 11D). At the transcript level, DEGs encoding CAT were down-regulated within 72 h of waterlogging stress compared with levels in controls (Figure 11E). The above results indicated that the three antioxidative enzymes in KR5 roots contributed to the response to waterlogging stress.

### 2.10. Identification of Unigenes or DEGs Encoding TFs

**Figure 12:**
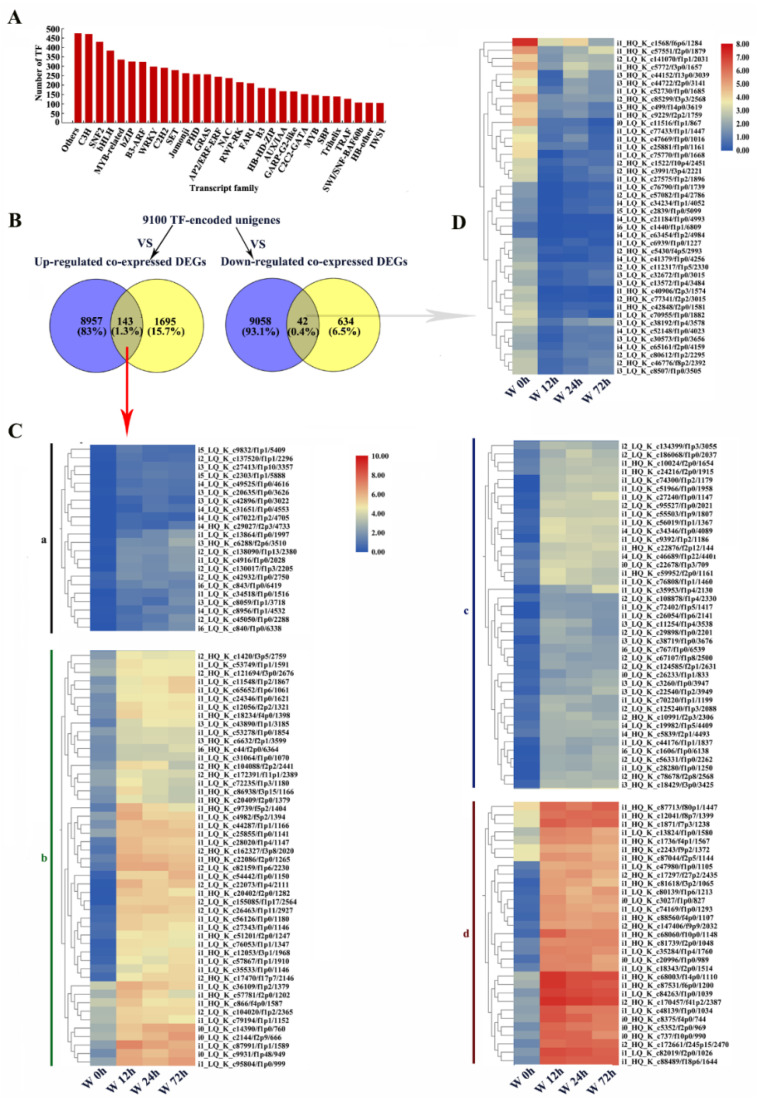
Identification of unigenes or DEGs encoding transcript factors (TF). (**A**) statistics of unigenes encoding TFs; (**B**) venn diagram of co-expressed DEGs and unigenes encoding TF; (**C**) a heatmap analysis of up-regulated co-expressed DEGs encoding TFs. According to expression patterns, these up-regulated co-expressed DEGs were clustered to 4 groups (a, b, c, d); (**D**) a heatmap analysis of down-regulated co-expressed DEGs encoding TFs. W, waterlogging.

In total, 9100 unigenes were predicted to be TFs (Table S4). The top 30 TF families included C3H, SNF2, bHLH, MYB-related, bZIP, B3-ARF, WRKY, C2H2 and AP2/ERF−ERF (Figure 12A). In pairwise comparison, between unigenes encoding TFs and co-expressed DEGs, 143 upregulated and 42 downregulated co-expressed DEGs were annotated as TFs (Figure 12B, Table S5). The upregulated TFs were further classified into 4 groups (a, b, c and d) based on their expression patterns under waterlogging stress (Figure 12C). In group d, the expression levels of DEGs increased significantly by waterlogging stress and these TFs were mostly in the LOB (10), AP2/ERF-ERF (8), Trihelix (5) and C3H (2) families (Figure 12C, Table S6). The downregulated TFs were mostly in the NF-YA (6), bHLH (5), MYB-related (5), C3H (3), AP2/ERF-ERF (2) and B3-ARF (2) families (Figure 12D). These results indicated that TFs played regulatory roles in adaptation to waterlogging stress in KR5 roots. 

### 2.11. WGCNA and Validation of Hub Genes by qRT-PCR

**Figure 13:**
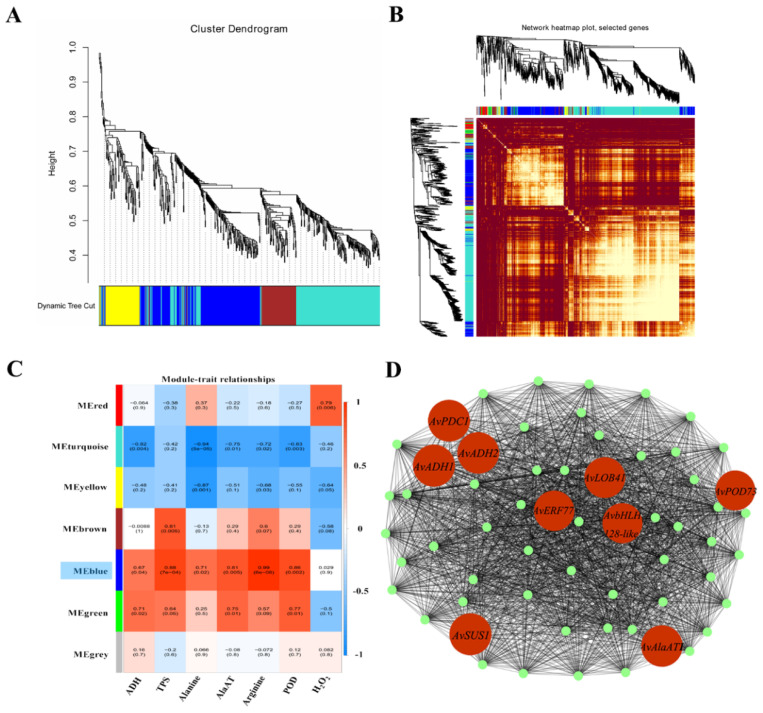
Identification of co-expression network modules. (**A**) cluster dendrogram of genes included in co-expression module analyses; (**B**) similarity of selected genes at the network topology level; (**C**) module-trait associations based on Pearson’s correlation coefficients; (**D**) gene network of the blue module, which was positively correlated with all selected indexes, except H_2_O_2_. Candidate genes within the blue module are highlighted in red and coded for gene descriptors based on annotations.

**Figure 14:**
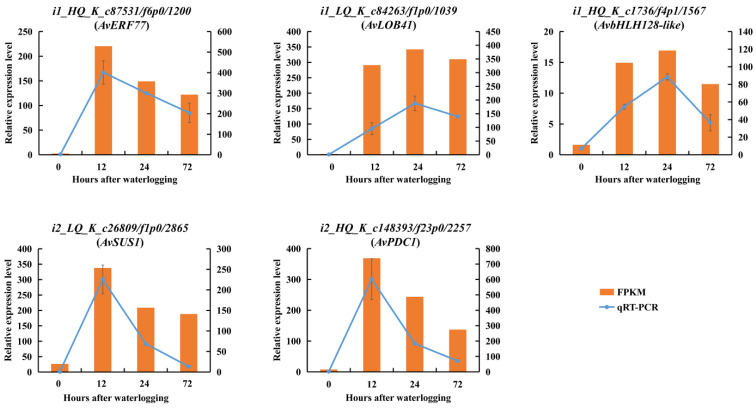
Expression patterns of several hub genes based on qRT-PCR (left vertical axis) and RNA-Seq (right vertical axis). Phylogenetic analyses of hub genes and their homologous genes were shown in Figure S6.

All DEGs were ranked by the sum of FPKM values from 12 independent samples (three replicates, four waterlogging durations). After the deletion of DEGs without annotation, the top DEGs with value above 500 were evaluated by WGCNA. Seven modules containing 40–1070 DEGs each were identified (Figure 13A, Table S7). The similarity of selected genes is shown at the network topology level (Figure 13B). The activity levels of ADH, TPS, AlaAT and POD and the contents of alanine, arginine and H_2_O_2_ were used as phenotypic data to calculate the relationships in trait modules (Figure 13C). In the blue module, the correlation coefficients ranged from 0.67 to 0.99, except for H_2_O_2_. We selected the top 60 hub genes in the blue module to construct a gene network (Figure 13D, Table S8). In this network, some hub genes involved in glycolysis and fermentation (ADH, PDC and SUS), alanine metabolism (AlaAT), ROS cleavage (POD) and genes encoding TFs (ERF, bHLH and LOB) were highly co-expressed. These results indicated that under the possible control of TFs, several genes associated with carbohydrate and amino acid metabolism and ROS cleavage were significantly upregulated in KR5 roots under waterlogging stress. In addition, the qRT-PCR results showed that expression patterns of the hub genes were similar to those observed in the RNA-Seq data, confirming the validity of the RNA-Seq results (Figure 6A,B, Figure 8D, Figure 11B and Figure 14). 

## 3. Discussion

In this study, the use of full-length libraries with long SMRT sequencing reads fa-cilitated the de novo transcriptome assembly of *A. valvata* roots. The N50 of the corrected transcripts using the PacBio RS II platform (3803 bp, Table S1) was higher than that of RNA-Seq transcripts (744 bp). The error rate of Pacbio can be overcome with correction of Illumina RNA-Seq and DEGs under treatments can be obtained using the Illumina platform. Via a hybrid sequencing approach, a more complete transcriptome was generated, which provided resources for investigating the waterlogging responses in *A. valvata* roots. Waterlogging stress threatens the growth of plants, especially in waterlogging-sensitive plants. Studying the response of waterlogging-tolerant genotypes will help in identifying key regulatory genes and metabolites associated with their higher waterlogging tolerance. The genotype KR5 from *A. valvata* was previously proven to be much more tolerant to waterlogging stress than other genotypes [31]. In this study, our results indicated that KR5 roots responded to waterlogging stress mainly by adjusting carbohydrate and amino acid metabolism and ROS cleavage.

Recent reports showed that lipid remodeling also played an important role in plant hypoxia stress [35,36,37,38,39]. In this study, several DEGs encoding LACS, SAD, AACT and LOX were significantly upregulated under waterlogging stress (Figure S3). LACS catalyzes the synthesis of long-chain acyl-CoAs and acyl-CoA thioesters [40]. Declines in cellular ATP levels under hypoxia conditions can lead to the decrease of LACS activity, which further causes the changes of the composition of the acyl-CoA pools [41]. Xie et al. found that LACS2-overexpressor lines possessed a higher tolerance to submergence [42]. Biosynthesis of long-chain acyl-CoA pools catalyzed by LACS2 regulates the intracellular trafficking and activity of subgroup VII ETHYLENE-RESPONSE FACTOR and modulates the cuticle permeability in Arabidopsis [42]. In KR5, LACS might be important for its acyl-CoA metabolism and waterlogging tolerance. SAD catalyzes the conversion of stearic acid (18:0) to oleic acid (18:1), which influences the cellular polyunsaturated fatty acid content and mediates membrane fluidity [43]. In ‘Zhemizhen 1’ (*A. polygama*), *AcSAD* was highly induced under waterlogging stress [44]. In *Arabidopsis*, transcript level of *AtSAD6* was increased under hypoxia stress [45]. In KR5, upregulation of *SADs* might be an important acclimation in response to waterlogging stress. AACT is involved in the biosynthesis of isoprenoids via mevalonate pathway [46]. In alfalfa, transcript level of *MsAACT1* is highly promoted in roots under cold and salinity stress [46]. In sandalwood (*Santalum album* L.), *SaAACT* genes were responsive to methyl jasmonate treatment [47]. Some ACATs are involved in the last step of fatty acid β-oxidation. In wheat, ACAT protein levels decreased in germinated seed under submergence stress [48]. In roots of KR5, isoprenoid metabolisms might be adjusted under waterlogging stress. LOX catalyzes the oxygenation of polyunsaturated fatty acids into oxylipins [49]. In *Glycine max*, *lipoxygenase 2* was specifically induced under flooding stress [50]. In *Zea maize*, LOX activity was significantly increased under waterlogging stress [51]. In *Arabidopsis*, expression levels of *LOXs* increased during post-submergence reoxygenation [52]. Taken together, lipid metabolisms may contribute to waterlogging responses in KR5. In the future, a lipidomic analysis and functional verification of genes related to lipid metabolisms are needed, which is useful for discerning the lipid composition changes and central genes in KR5 under waterlogging stress.

### 3.1. Adjustments of Carbohydrate Metabolisms under Waterlogging Stress

The activation of glycolysis and fermentation is a key feature for acclimation to hypoxia. In this study, many well-known hypoxia-related genes, including *SUS*, *PGI*, *ADH* and *PDC*, were induced in KR5 roots under waterlogging stress (Figure 4 and Figure 14). SUS is a key enzyme involved in sucrose degradation under hypoxic conditions. Sucrose metabolism by the sucrose synthase pathway consumes less energy than the invertase pathway [53]. A sufficient carbohydrate reserve fuels glycolysis. In the current study, the increase in the sucrose content in KR5 roots after 72 h of waterlogging might be important for survival under a longer period of waterlogging stress. PGI is required for glycolysis. Increased transcript levels and enzyme activity of PGI have been observed in waterlogged roots [14,54]. Ethanolic fermentation provides NAD^+^ to maintain glycolysis. PDC and ADH play essential roles in plant tolerance to hypoxia. The overexpression of kiwifruit *PDC* and *ADH* confers enhanced waterlogging tolerance in *Arabidopsis* [55,56,57]. TPS is the key enzyme in the synthesis of T6P, which acts as a signaling molecule and regulates plant responses to diverse stresses [58]. In the current study, the significant increase in TPS activity and increased expression level of a *TPS* encoded DEG indicated that trehalose metabolism contributed to the response to waterlogging stress in KR5 roots. Energy-consuming biosynthesis processes are usually downregulated under low-oxygen conditions [59]. Genes associated with starch and cellulose metabolism are affected by waterlogging stress in many plants [60,61]. In this study, at the transcript level, the expression of co-expressed DEGs encoding enzymes associated with cellulose and starch metabolism was downregulated (Figure 4). Beta-glucosidase is an enzyme involved in cellulose hydrolysis. The glucose product can inhibit the reaction catalyzed by beta-glucosidase [62]. It can be deduced that the significant increase in beta-glucosidase activity after 24 h of waterlogging stress was partly due to the dynamic changes in glucose. 

### 3.2. Adjustments of Free Amino Acid Metabolism under Waterlogging Stress

Waterlogging stress alters the levels of amino acids in plants [14,63]. Our data showed that the accumulation of total free amino acids in KR5 roots was among the major metabolic responses to waterlogging stress (Figure 7A). Protein turnover can contribute to an increase in the free amino acids [64]. Amino acids play a role in osmotic adjustment and the synthesis of secondary metabolites, mitigating the adverse effects of abiotic stresses [65,66]. In *Arabidopsis*, under hypoxic conditions, most accumulated amino acids are derived from pyruvate or intermediates of glycolysis [67]. In the KR5 roots, an overall up-regulation of amino acids in the pyruvate family (e.g., alanine) was observed (Figure 7B). It has been proposed that, the inhibition of aerobic respiration decreases flux into the tricarboxylic acid (TCA) cycle (e.g., aspartate), leading to a redirection of carbon to amino acids derived from glycolysis intermediates (e.g., alanine) under hypoxic stress [14,68,69]. The direction of pyruvate to alanine, rather than ethanolic fermentation, can prevent the excess accumulation of ethanol and lactate as well as the carbon loss under hypoxic conditions [70]. In rice, the glutamine synthetase (GS)/NADH-GOGAT cycle is required for the anaerobic accumulation of alanine [71]. In legume species, the main route of alanine synthesis under hypoxia is the interconversion of pyruvate and glutamate to alanine and 2-oxoglutarate, which is catalyzed by AlaAT [68,69]. In this study, following the increases in glutamate and alanine, the enzyme activities and gene expression levels of NADH-GOGAT and AlaAT were elevated in KR5 roots under waterlogging stress (Figure 8 and Figure 9). Our results suggested that NADH-GOGAT/AlaAT cycle played a role in the concerted adjustment of alanine and glutamate metabolism in KR5 roots. Glutamate-derived amino acids were the most abundant in the KR5 roots. In the glutamate family, the arginine content increased in KR5 roots under waterlogging stress. Arginine metabolism play a crucial role in nitrogen distribution and recycling in plants [72]. Nitrogen metabolism was affected by waterlogging stress in KR5 roots.

### 3.3. Adjustments of ROS Metabolism under Waterlogging Stress

Levels of superoxide anion and H_2_O_2_ increased after 24 or 72 h of waterlogging stress, indicating that oxidative injury in KR5 roots were more severe at later stages of waterlogging. Antioxidant systems were boosted in KR5 waterlogged roots by increasing POD and CAT activity, rather than SOD activity (Figure 10C and Figure 11C,D). The reduced activity of SOD might lead to an increase in the superoxide anion content after 24 h of waterlogging. A decrease in SOD activity has also been observed in corn and mung bean under flooding conditions [73,74]. The intraorganellar distribution and/or enzyme characteristics related to the functional differences of each SOD isoform in response to salt stress have been reported [26]. In the current study, the expression levels of *Mn-SODs* were specifically increased under waterlogging stress (Figure 10D,E). POD and CAT are involved in the regulation of H_2_O_2_ levels [75]. In this study, POD and CAT efficiently removed H_2_O_2_ after 12 and 24 h of waterlogging, highlighting their major roles in the regulation of ROS homeostasis. Enhanced POD activity can help plants adapt to flooding stress [76]. In the present study, consistently high POD activity increased the ROS scavenging efficiency in KR5 roots, leading to waterlogging tolerance. Along with the high enzyme activity, however, the expression levels of DEGs encoding POD and CAT were mostly downregulated (Figure 11A,E). Taken together, ROS detoxification was achieved by adjusting antioxidative enzymes at both the protein and mRNA levels in KR5 roots under waterlogging stress. 

### 3.4. Effects of TFs on the Regulation of Waterlogging-Responsive Genes

TFs play crucial roles in the regulation of the response of plants to various abiotic stresses [77]. In this study, genes in the LOB, Trihelix and C3H zinc finger TF families were also greatly induced under waterlogging stress (Figure 12C). The expression levels of LOB domain (LBD) family genes were increased under flooding stress in *Solanum dulcamara* and *Arabidopsis* [78,79]. In maize, the mRNA expression levels of 14 of 59 Trihelix TFs increased under waterlogging stress [80]. Genes encoding zinc-finger proteins were significantly upregulated in *Zea maize* and rice under hypoxic stress [81,82]. The detailed mechanisms by which different TFs function in response to waterlogging stress in kiwifruit require further study.

### 3.5. WGCNA of the Gene Regulatory Network for the Response to Waterlogging Stress

Plants initiate complex transcriptional and metabolic responses under waterlogging stress. Some waterlogging-responsive genes show coordinated regulation under the possible control of TFs [83]. In this study, by WGCNA, one module (blue) was strongly correlated with the majority of selected phenotypic traits (Figure 13C). In the blue module, the hub genes *SUS*, *PDC* and *ADH* are associated with sucrose metabolism and fermentation, *AlaATs* are associated with alanine metabolism, and *PODs* are associated with ROS cleavage (Figure 13D). ERF, bHLH and LBD41 are important regulators of waterlogging stress. In line with our KEGG pathway enrichment results, the WGCNA results also suggested that carbohydrate and amino acid metabolism and ROS detoxification were primary processes in the response of KR5 roots to waterlogging stress. In the future, the exact relationships between these hub genes need to be explored to further improve our understanding of the mechanism underlying waterlogging tolerance in kiwifruit.

## 4. Materials and Methods

### 4.1. Plant Materials and Waterlogging Treatments

The genotype KR5 (*A. valvata*) is highly tolerant to waterlogging stress [31]. In this study, asexually propagated KR5 plants were used as the materials. Plants were normally managed in a greenhouse at 25–28 °C. Healthy potted plants (18-cm-diameter pot), at the five- to six-leaf stage, were selected for waterlogging treatments. Two pots were placed in a plastic container (45 cm × 35 cm × 16 cm) filled with water and the water level was maintained at 2–3 cm above the soil surface. The plants were subjected to waterlogging stress for 0, 12, 24 and 72 h. No waterlogging (i.e., 0 h) was used as a control. 

### 4.2. RNA Extraction, Illumina Library Construction, Sequencing and De Novo Assembly

Root samples from 12 plants (three replicates, four plants per replicate) were quickly collected after 0, 12, 24 and 72 h of waterlogging and immediately frozen in liquid nitrogen. After complete sample homogenization, total RNA was extracted using the RNeasy Mini Kit (Qiagen, Germantown, MD, USA). Total RNA was quantified using an Agilent 2100 Bioanalyzer (Agilent Technologies, Santa Clara, CA, USA), NanoDrop 2000 spectrophotometer (Thermo Fisher Scientific, Santa Clara, CA, USA) and 1% agarose gel. Approximately 1 μg of total RNA with a RIN value above 7 was used for library preparation.

Libraries were constructed using the NEBNext^®^ Ultra™ RNA Library Prep Kit for Illumina (San Diego, CA, USA), according to the manufacturer’s protocol and then the multiplexed libraries were loaded on an Illumina HiSeq2000 instrument. Sequencing was carried out based on a 2 × 150-bp paired-end configuration. Technical sequences were removed using Cutadapt (version 1.9.1, TU Dortmund University, TU Dortmund, Germany). The trimmed reads were assembled using Trinity (r20140413p1, Massachusetts Institute of Technology, Cambridge, MA, USA) and unigene sequences were obtained after removing duplicated contigs using cd-hit. Illumina sequencing data were deposited in the NCBI GEO database under the accession number PRJNA792211.

### 4.3. PacBio Library Construction, Sequencing and Preprocessing

To construct the PacBio Iso-Seq library, total RNA from the whole KR5 plant was pooled from all treatments (i.e., four time periods of waterlogging) in equal quantities, including plant samples from 12 independent biological replicates. The library was constructed in accordance with the standard PacBio Iso-Seq sequencing protocol and then sequenced on the PacBio Sequel platform (Allwegene, Beijing, China). The raw data were processed to remove the connector and low-quality reads by using SMRTlink v6.0 (parameters: -minLength 50, -maxLength 1500, -minPasses 2, -min_seq_len 200, Pacific Biosciences of California, Inc, Menlo Park, CA, USA). After self-correction using the circular consensus sequence (CCS) algorithm, the CCS sequence was obtained. CCSs were classified into full-length non-chimeric (FLNC) and non-full-length non-chimeric (nFL) sequences. Iterative clustering for error correction (ICE) was used to cluster the FLNC subreads that belonged to the same transcript. The clustered consensus subreads were polished with nFL to obtain high-quality polished consensus sequences using Arrow. The PacBio Iso-Seq FL transcriptome data were deposited in NCBI databases under the accession number PRJNA796628. The RNA-Seq data sequenced from KR5 (*A. valvata*) roots waterlogged for 0, 12, 24 and 72 h were used for validation of the polished consensus sequence using LoRDEC V0.7 (parameters: -k 23; -s 3, CNRS and Université Montpellier, Montpellier, France). The corrected transcript sequences were obtained by removing redundancies using CD-Hit V4.6.8 (parameters: -c 0.95, -T 6, -G 0, -aL 0.00, -aS 0.99, -AS 30). The core conserved gene set of Eukaryota and BUSCO (version: 3.0.2) were used to evaluate the assembly quality of the dataset of the full-length transcriptome [84].

### 4.4. Functional Annotation of Transcripts and Prediction of CDSs and TFs

BLAST was used to annotate the unigenes by searches against the NCBI non-redundant (NR) protein database, Swiss-Prot database, KEGG database, eukaryotic ortholog groups (KOG) database, GO database, NCBI nucleotide sequences (NT) database and the protein family (Pfam) database. GO-Term Finder was used to identify Go terms associated with a group of enriched genes with *p* < 0.05. Significant DEGs were evaluated by a KEGG pathway analysis using in-house scripts. Coding sequence (CDS) prediction was performed using ANGEL v. 2.4 (parameters: --min_angel_aa_length 50, error tolerant mode, Pacific Biosciences of California, Inc., Menlo Park, CA, USA). iTAK: 1.7a was used to predict the plant TFs (parameters: -f 3F).

### 4.5. Identification of DEGs and qRT-PCR Analysis

The unigene sequences obtained by RNA-Seq were aligned to the corrected transcript sequences obtained by SMRT sequencing, regarded as a reference. The DESeq Bioconductor package was used for a differential expression analysis. After adjustments using the Benjamini and Hochberg approach to control for the false discovery rate (FDR), we set a *p* ≤ 0.05 and absolute value of log2 (fold-change) ≥ 1 as the thresholds for determining DEGs. 

For the qRT-PCR analysis, cDNA was synthesized using a ReverTra Ace qPCR RT kit (TOYOBO, Osaka, Japan) according to the manufacturer’s protocol. The reactions were performed using LightCycler 480 SYBR Green I Master Mix. A melting curve analysis was conducted to validate the amplification specificity of each run. Expression was normalized against levels of the kiwifruit actin gene. The sequences of primers used for amplification are listed in Supplementary Table S9. Heatmaps were generated using the software TBtools (a toolkit for biologists integrating various biological data-handling tools) [85]. The heatmap was constructed based on average FPKM expression values (three replicates) of DEGs.

### 4.6. Sucrose, T6P, H_2_O_2_ and Superoxide Anion Content Measurements

Sucrose was extracted from finely chopped tissue twice using 80% *v/v* ethanol at 80 °C. After the dilution of pooled sucrose extract, the sucrose content was measured using the resorcinol-hydrochloric acid colorimetric method according to a previously described procedure [86]. The T6P content was measured using a commercial ELISA kit (SINOBESTBIO, Shanghai, China). The H_2_O_2_ content was measured using the titanic sulfate method as described previously [87]. The superoxide radical content was determined according to a previously described method [88]. 

### 4.7. ADH, TPS, Beta-Glucoside, AlaAT, NADH-GOGAT, SOD, POD and CAT Activity

ADH was extracted following a previously described method [89]. ADH activity was measured following a previously described protocol [90]. A two-step reaction method was used for the TPS activity assay, according to a previously described protocol [91]. The activity of β-glucoside was assayed using commercial kits (Suzhou Michy Biomedical Technology Co., Ltd., Suzhou, China). AlaAT was extracted and assayed according to a previously described method [92]. The activity of NADH-GOGAT was assayed using commercial kits (Suzhou Michy Biomedical Technology Co., Ltd., Suzhou, China). SOD, POD and CAT were extracted using a precooled sodium phosphate buffer solution (50 mM, pH 7.0). SOD activity was measured by recording the reduction rate of nitro blue tetrazolium chloride at an absorbance of 560 nm [89]. POD activity was measured using the guaiacol method, according to a previously described procedure [89]. CAT activity was measured by monitoring the decrease in H_2_O_2_ at 240 nm for 1 min [93]. The protein contents of the enzyme extracts were determined using the Bradford method [94].

### 4.8. Free Amino Acid Content Measurements

The free amino acid content was determined by a previously described pre-column derivatization RP-HPLC method [95], with a slight modifications. For hydrolysis, approximately 0.1 g of sample was mixed with hydrochloric acid (0.1 M, 1 mL) and vigorously vortexed for 60 s, after which the sample was left to stand at 4 °C overnight. Then, the tube contents were centrifuged, and the supernatant was membrane-filtered to obtain the hydrolysate. For derivatization, a standard amino acid solution or sample hydrolysate (200 μL) was placed into a 2 mL tube and 20 μL of internal standard dl-norleucine solution was added. Next, 100 μL of triethylamine-acetonitrile (2.8:17.2, *v*/*v*) and 100 μL of phenylisothiocyanate-acetonitrile (0.125:10, *v*/*v*) were added to each tube, mixed and incubated for 1 h at 25 °C. Next, 400 μL of n-hexane was added to each tube, mixed and placed at 25 °C for 10 min. The resulting lower liquid was filtered and then used for detection. Chromatographic analysis was carried out using a RIGOL L-3000 HPLC system. The Amethyst C18-H column (250 mm × 4.6 mm, 5 μm) was maintained at 40 °C. The flow rate was set at 1.0 mL min^−1^ and the injection volume was 10 μL. The measurements were obtained at a wavelength of 254 nm. 

### 4.9. Statistics Analyses and WGCNA 

Statistical analyses were conducted using SPSS Statistics (version 22.0). After analysis of variance (ANOVA), significant differences were determined using Duncan’s multiple range tests (*p* < 0.05). WGCNA was preformed to identify the correlations between gene expression profiles and phenotypic traits using the WGCNA package in R (version 1.69). Modules were obtained using an automatic network construction function with default settings. Visualization was performed using Cytoscape (version 3.8.2, Bethesda, MD, USA).

## 5. Conclusions

Full-length unigenes of a waterlogging-tolerant kiwifruit rootstock KR5 (*A. valvata*) were obtained by combining de novo sequencing with PacBio Iso-Seq. KR5 roots responded to waterlogging stress by adjusting carbohydrate and amino acid metabolism and cleaving ROS. We identified several waterlogging-related genes and characterized the dynamic changes in correlated enzymes and metabolites. The WGCNA results revealed a candidate gene co-expression network related to waterlogging responses. Overall, our results expand our understanding of the waterlogging-induced regulatory mechanisms in a waterlogging-tolerant kiwifruit genotype. In the future, specific adaptation strategies that render KR5 more tolerant to waterlogging than other genotypes need to be explored and the functions of several candidate genes need to be verified. 

## Data Availability

The raw data generated in this study have been deposited in the NCBI Short Read Archive (SRA) under the accession number. All other relevant data were included in the paper and Supplementary Materials.

## Supplementary Materials

The following supporting information can be downloaded at: https://www.mdpi.com/article/10.3390/ijms23063237/s1.
