# Supplementary material for: Full-Length Transcriptome and RNA-Seq Analyses Reveal the Mechanisms Underlying Waterlogging Tolerance in Kiwifruit (Actinidia valvata)
Source: Int J Mol Sci. 2022 Mar 17;23(6):3237. doi: 10.3390/ijms23063237 (PMC8951935; doi:10.3390/ijms23063237)
Supplement: Supplementary file 1 [file ijms-23-03237-s001.zip › Supplemental figure legends and table captions.pdf]

Supplementary Materials:

Additional file 1:

Supplementary Figure S1 BUSCO assessment results of full-length transcriptome.

Additional file 2:

Supplementary Figure S2 Annotation and GO functional classification of the unigenes. A, number of unigenes annotated by public databases; B, venn analysis of the number of annotated unigenes by public databases; C, GO functional classification of unigenes.

Additional file 3:

Supplementary Figure S3 A heatmap analysis of co-expressed upregulated DEGs involved in the fatty acid and alpha-linolenic acid metabolisms pathways. Expression levels of genes shown in the shade box were more highly promoted under waterlogging stress.

Additional file 4:

Supplementary Figure S4 Heatmaps of co-expressed upregulated (A) and downregulated (B) DEGs involved in the 'starch and sucrose metabolism' pathway.

Additional file 5:

Supplementary Figure S5 Heatmaps of DEGs encoding alcohol dehydrogenase (ADH) under waterlogging stress. A, *ADH1*; B, *ADH2*. Asterisks indicate unigenes whose expression levels were the most highly upregulated under waterlogging stress in the *ADH1* and *ADH2*, respectively.

Additional file 6:

Supplementary Figure S6 Phylogenetic tree of candidate genes in response to waterlogging stress and their homologous genes. A, ADH1 and ADH2; B, PDC1; C, SUS1; D, AlaAT1; E, POD73; F, LOB41; G, bHLH128-like.

Additional file 7:

Table S1. Summary of the transcriptome data for KR5 (*A. valvata*) using PacBio Iso-Seq.

Table S2. Summary of reads sequenced from KR5 roots using Illumina RNA-Seq.

Additional file 8:

Table S3. Changes in free amino acid contents in KR5 roots under waterlogging stress.

Additional file 9:

Table S4. Unigenes predicted to encode transcription factors (TFs).

Table S5. Co-expressed DEGs predicted to encode TFs.

Table S6. Members of the d group from upregulated co-expressed TFs.

Additional file 10:

Table S7. Genes enriched in the seven modules obtained by WGCNA.

Table S8. Top 60 hub genes in the blue module obtained by WGCNA.

Additional file 11:

Table S9. Primers used for qRT-PCR.
