# Supplementary figures and images for: Full-Length Transcriptome and RNA-Seq Analyses Reveal the Mechanisms Underlying Waterlogging Tolerance in Kiwifruit (Actinidia valvata)
Source: Int J Mol Sci. 2022 Mar 17;23(6):3237. doi: 10.3390/ijms23063237 (PMC8951935; doi:10.3390/ijms23063237)

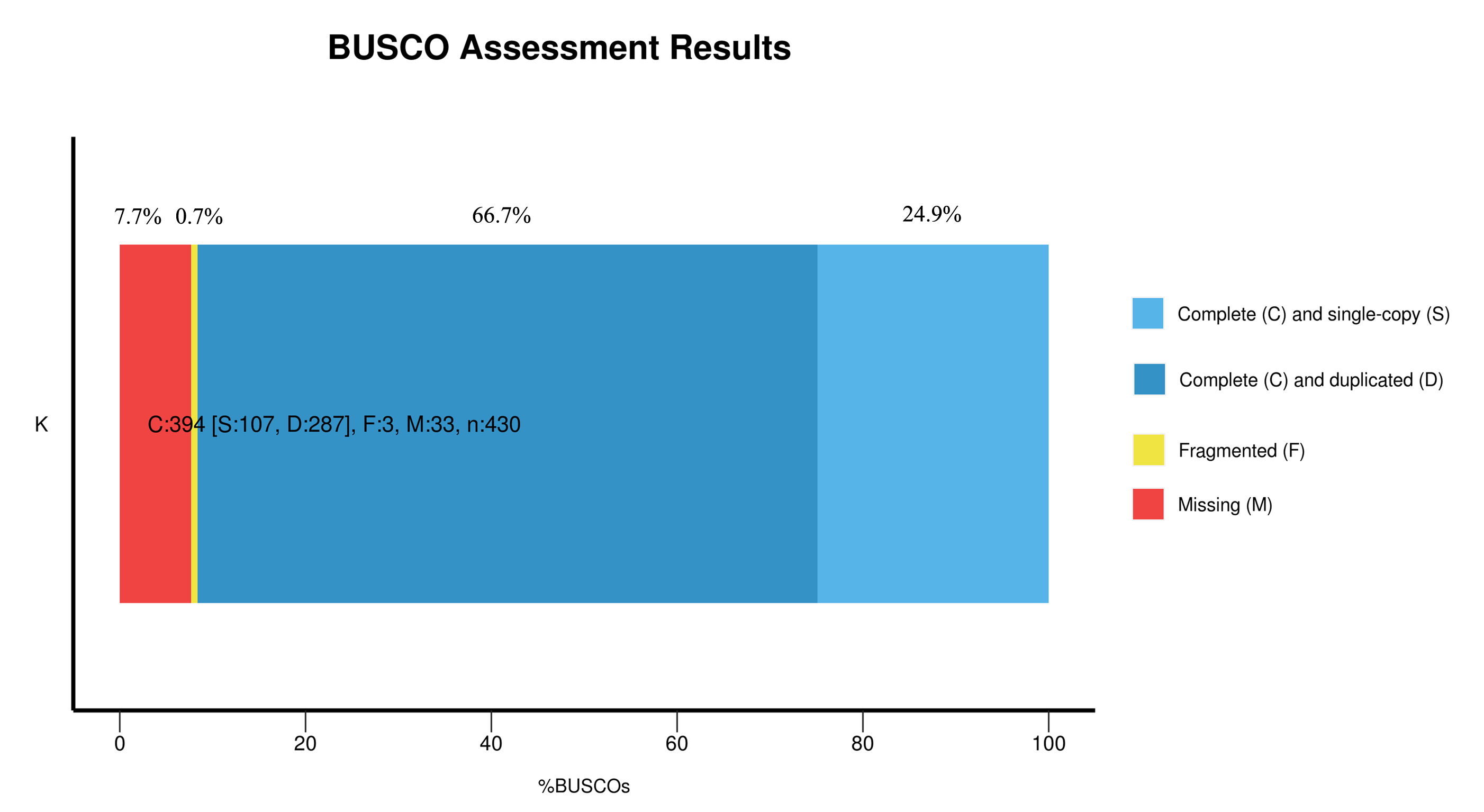

Supplement: Supplementary file 1 [file ijms-23-03237-s001.zip › Additional file 1.tif]

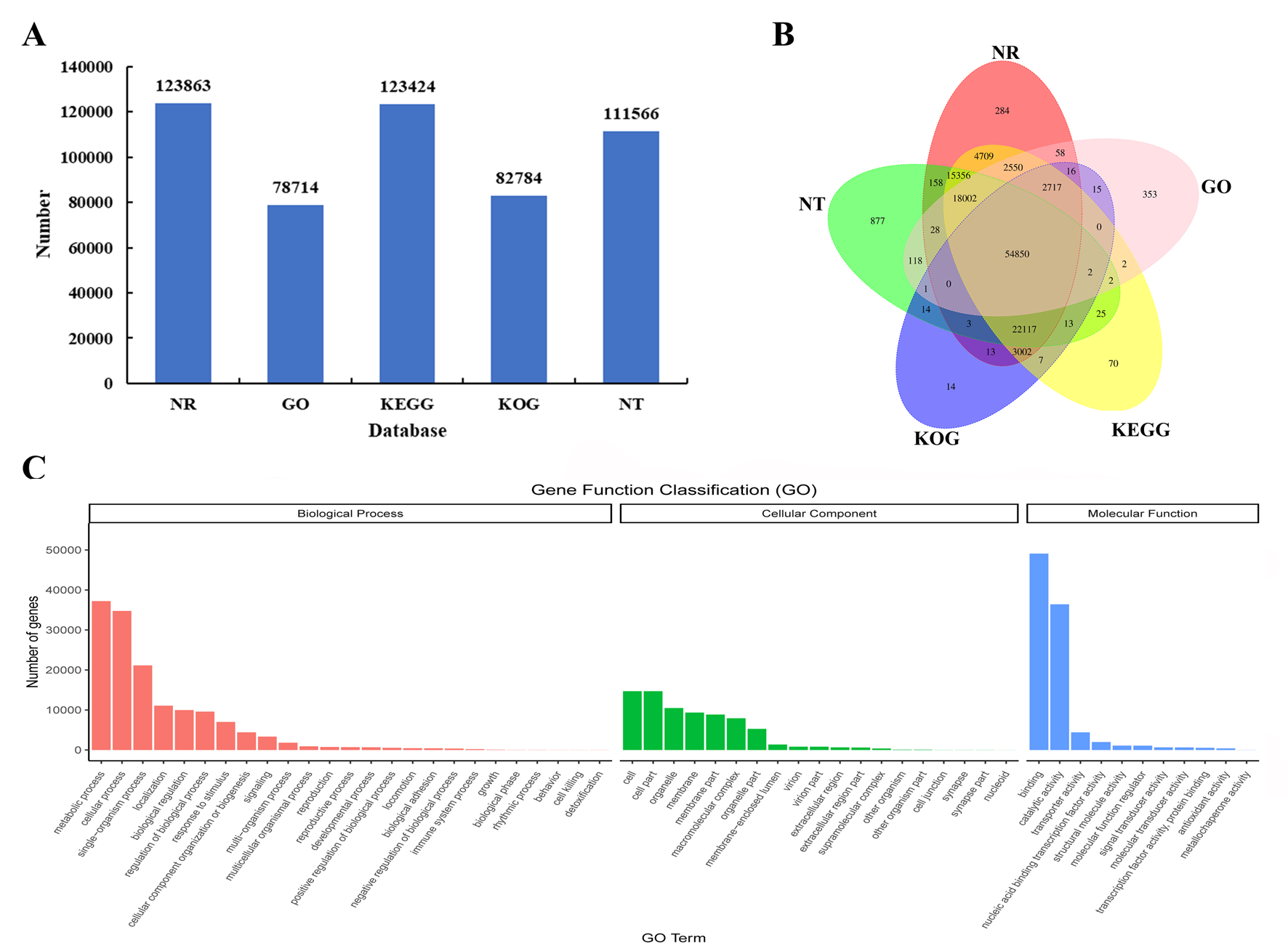

Supplement: Supplementary file 1 [file ijms-23-03237-s001.zip › Additional file 2.tif]

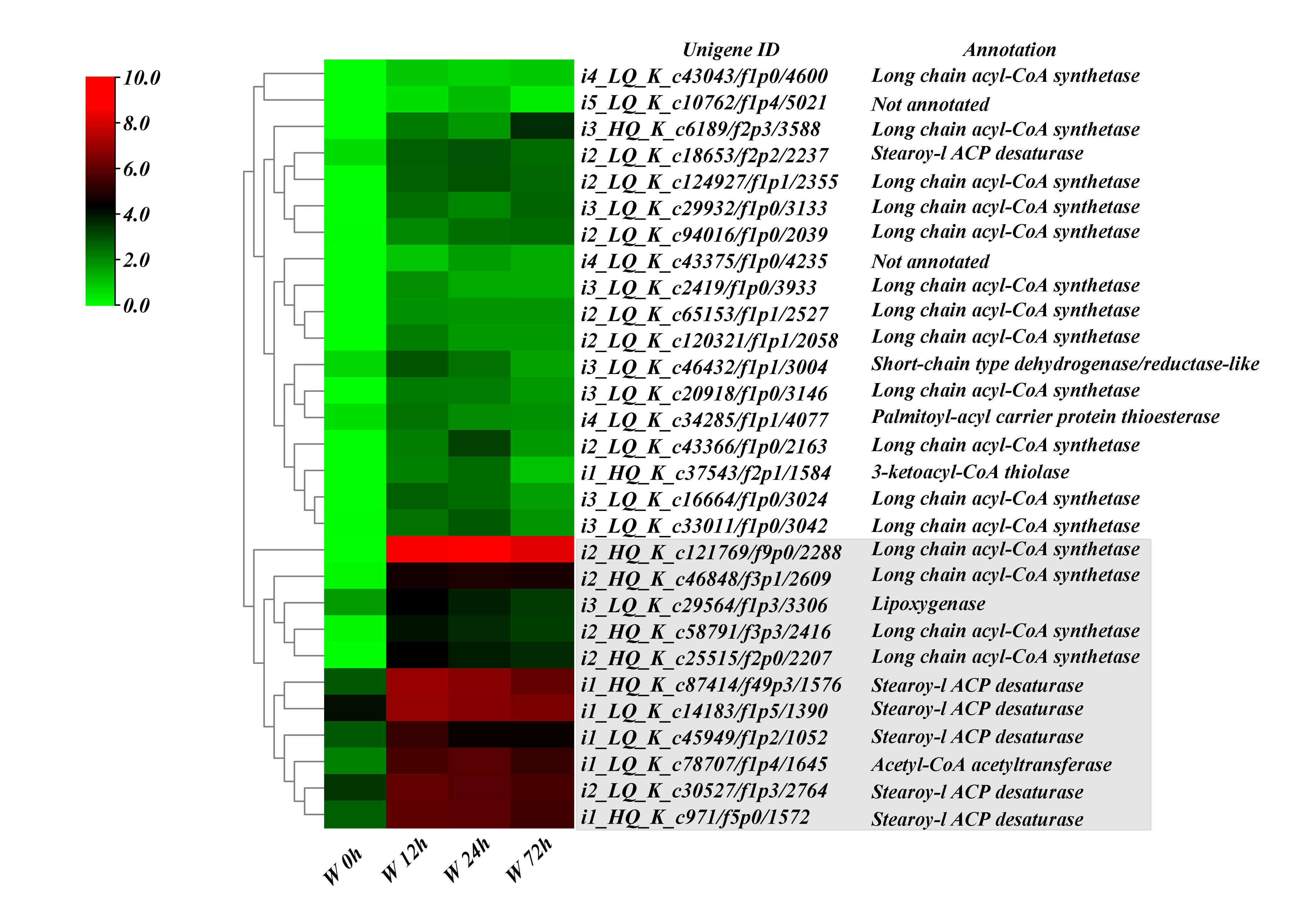

Supplement: Supplementary file 1 [file ijms-23-03237-s001.zip › Additional file 3.tif]

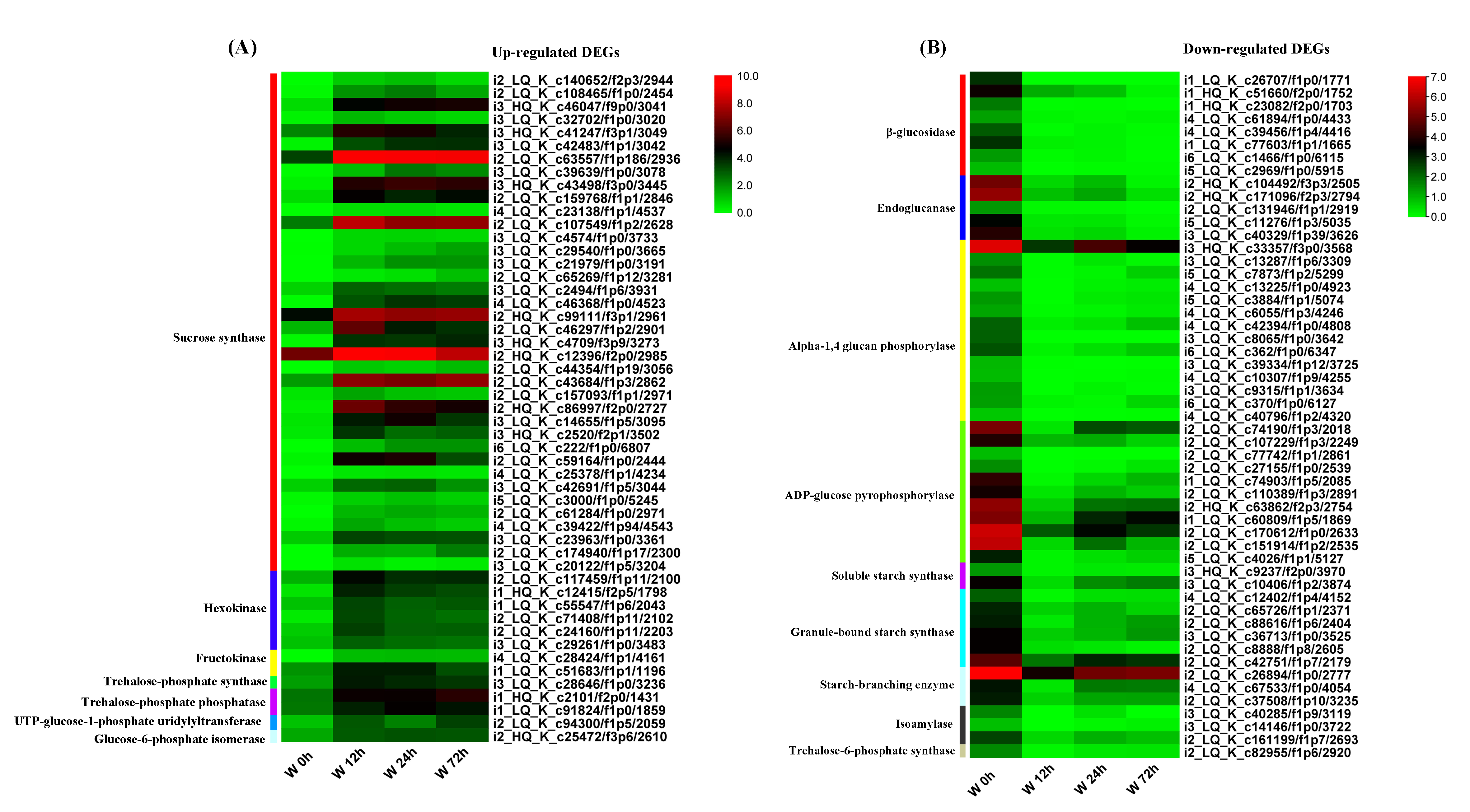

Supplement: Supplementary file 1 [file ijms-23-03237-s001.zip › Additional file 4.tif]

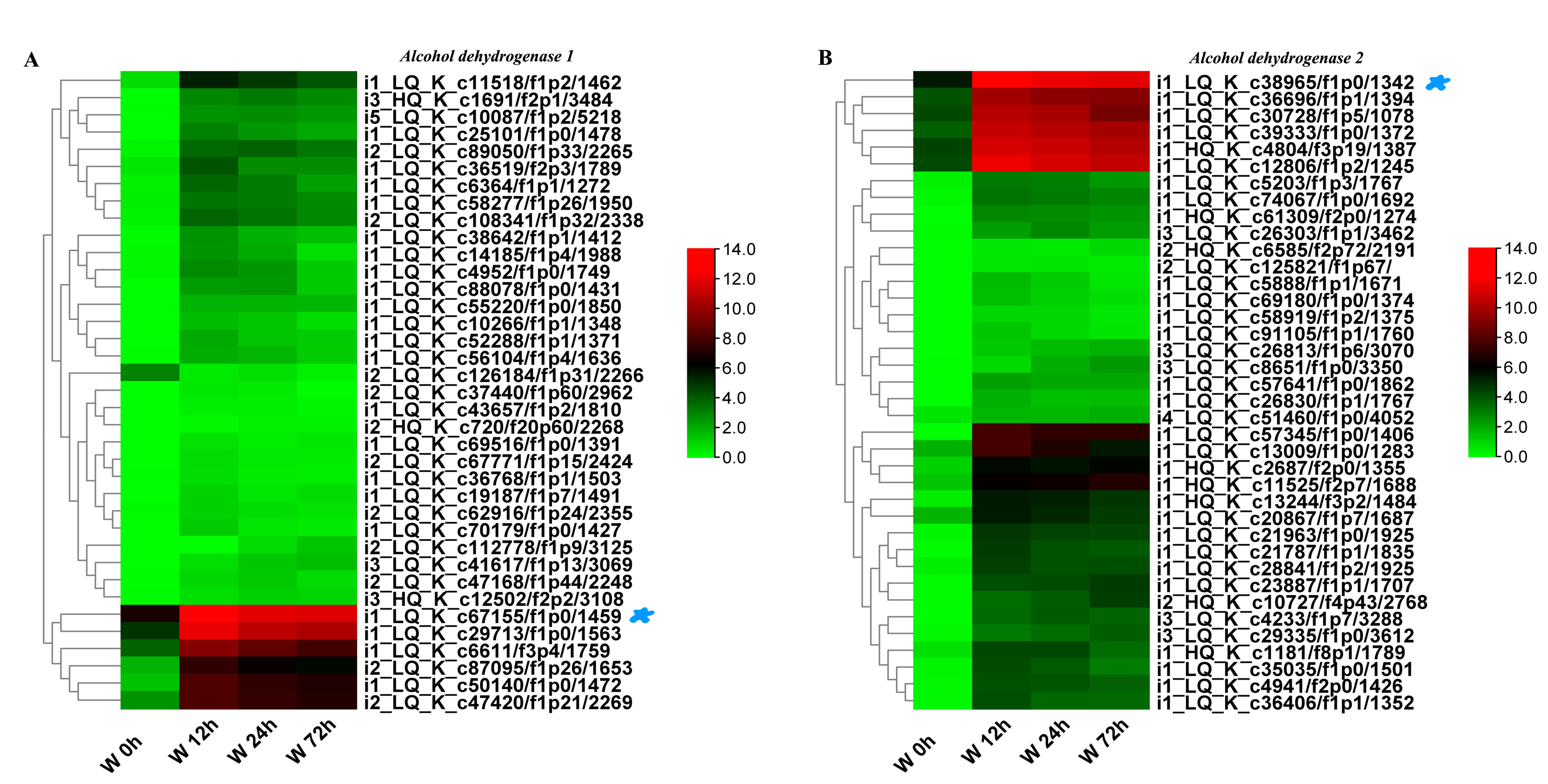

Supplement: Supplementary file 1 [file ijms-23-03237-s001.zip › Additional file 5.tif]

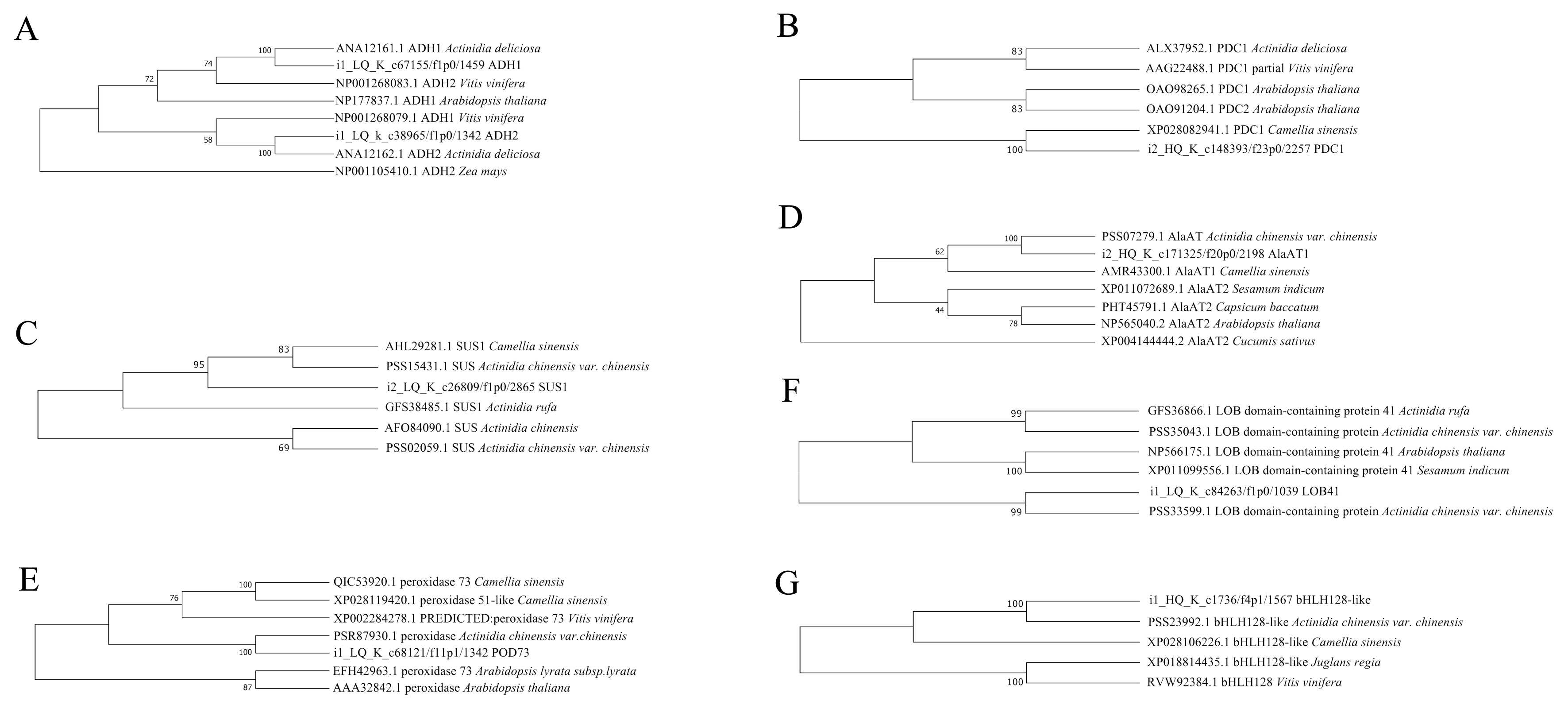

Supplement: Supplementary file 1 [file ijms-23-03237-s001.zip › Additional file 6.tif]
